# Probiotics ameliorate IgA nephropathy by improving gut dysbiosis and blunting NLRP3 signaling

**DOI:** 10.1186/s12967-022-03585-3

**Published:** 2022-08-29

**Authors:** Jiaxing Tan, Lingqiu Dong, Zheng Jiang, Li Tan, Xinyao Luo, Gaiqin Pei, Aiya Qin, Zhengxia Zhong, Xiang Liu, Yi Tang, Wei Qin

**Affiliations:** Division of Nephrology, Department of Medicine, West China Hospital, Sichuan University, Chengdu, Sichuan China

**Keywords:** IgA nephropathy, Gut dysbiosis, Probiotics, Short-chain fatty acid, NLRP3

## Abstract

**Background:**

Recently, a few studies have indicated a relationship between the gut microbiota and IgA nephropathy (IgAN). Whether the gut microbiota participates in the pathogenesis of IgAN and whether probiotics are effective in treating IgAN are still controversial. Therefore, this study aimed to identify the differences in the structure of the gut microbiota between IgAN and controls and to evaluate the efficacy and mechanism of probiotics in the treatment of IgAN.

**Methods:**

To address this question, 35 IgAN patients and 25 healthy volunteers were enrolled, and a mouse IgAN model was also constructed. The stool microbes were analyzed by 16S rRNA high-throughput sequencing to identify the differential strains between IgAN and healthy controls. The impact of probiotics on the structure of the intestinal flora and the efficacy of the probiotics in the treatment of IgAN were evaluated.

**Results:**

Although the microflora structure of mice and humans was not the same, both patients and mice with IgAN exhibited gut microbiota dysbiosis, with all subjects presenting an evident decrease in *Bifidobacterium* levels. The *Bifidobacterium* proportion was negatively correlated with proteinuria and hematuria levels, indicating that the decreased *Bifidobacterium* abundance could be related to IgAN severity. Probiotic treatment containing *Bifidobacterium* in IgAN mice could significantly alleviate gut dysbiosis, specifically by increasing the proportion of beneficial bacteria and reducing the abundance of potentially pathogenic bacteria. Moreover, both probiotics and their metabolites, short-chain fatty acids (SCFAs), could attenuate IgAN clinicopathological manifestations by inhibiting the NLRP3/ASC/Caspase 1 signaling pathway.

**Conclusions:**

Supplementation with probiotics mainly containing *Bifidobacterium* could markedly improve gut dysbiosis in IgAN. Moreover, both probiotics and their SCFA metabolites could attenuate the clinicopathological manifestations of IgAN by inhibiting the NLRP3/ASC/Caspase 1 signaling pathway. Therefore, probiotics have potential as an adjunctive therapy for IgAN.

**Supplementary Information:**

The online version contains supplementary material available at 10.1186/s12967-022-03585-3.

## Introduction

Although the pathogenesis of IgA nephropathy (IgAN) is not fully understood, the majority of scholars recognize that IgAN is an immune system disease caused by galactose-deficient IgA1 (Gd-IgA1) [1–3]. Emerging studies have indicated that gut-associated lymphoid tissue (GALT) and gut microbiota might be associated with the pathogenesis of IgAN since GALT and gut microbiota could be important sites for the production of Gd-IgA1 [4–6]. Intestinal bacteria and their metabolites have important impacts on the homeostasis of the gut mucosal immune network. An increasing number of studies have proven that gut microbes are crucial for the host immune response and keep the immune system well-balanced [5, 7]. Modern perspectives believe that changes in the composition of the gut microbiota could alter systemic or local mucosal immune responses and might be involved in the pathogenesis of IgAN [7–9].

*Bifidobacterium spp.* are the most important probiotics in the human body and play crucial roles in preventing pathogen invasion, maintaining mucosal homeostasis, strengthening gut integrity and regulating host immunity [10, 11]. Additionally, *Bifidobacterium spp.* can ferment sugars to produce endogenous short-chain fatty acids (SCFAs) that are volatile saturated fatty acids with no more than 6 carbon atoms in the aliphatic chain. The most abundant SCFAs present in the human colon are sodium acetate (SA) and sodium propionate (SP) [12–14]. As metabolites of probiotics, SCFAs have been reported to have a positive impact on the host’s energy balance, lipid metabolism and immune regulation, and recent studies have illustrated that SCFAs could have renoprotective effects, but the mechanism has not been fully elucidated [13, 15–17].

With further in-depth research, gut dysbiosis has been shown to be associated with chronic kidney disease (CKD) and diabetic nephropathy. Several studies have reported that probiotics could have adjuvant therapeutic effects on patients with kidney disease [18–20]. Taking an adequate dose of probiotics can improve gut dysbiosis and increase the levels of SCFAs [13, 21]. However, the specific efficacy and mechanism of probiotics in the treatment of kidney disease remain unknown.

The NOD-like receptor family pyrin domain containing 3 (NLRP3) is a multiprotein complex that can activate apoptosis-associated speck-like protein (ASC) and Caspase 1 and regulate the formation of interleukin-18 (IL-18) and interleukin-1β (IL-1β), causing an inflammatory cascade [22]. Many scholars have proposed that the NLRP3 inflammasome plays an important role in the development of IgAN [23]. Interestingly, although research on the mechanism of probiotics in the treatment of kidney diseases is still lacking, several studies have reported that probiotics, especially *Bifidobacterium spp.*, could inhibit the expression of the NLRP3 gene. Sheng et al. treated ulcerative colitis mice with *Bifidobacterium spp.* and found that *Bifidobacterium spp.* could reduce the disease activity index, down-regulate the levels of IL-1β, and inhibit the mRNA expression of NLRP3 [24]. Yan et al. found that *Bifidobacterium spp.* can exert anti-inflammatory effects by inhibiting the expression of NLRP3 and ASC, thereby reducing liver damage caused by a high-fat diet [25]. In addition, *Bifidobacterium spp.* can also alleviate visceral hypersensitivity in irritable bowel syndrome by inhibiting NLRP3 [26]. These findings suggest that *Bifidobacterium spp.* may exert probiotic effects by inhibiting the NLRP3 signaling pathway. However, whether probiotics can treat IgA nephropathy by inhibiting the NLRP3 signaling pathway is still unclear.

Recently, a few studies have indicated a relationship between the gut microbiota and IgAN [8, 27]. Whether the gut microbiota participates in the pathogenesis of IgAN and whether probiotics are effective in treating IgAN are still controversial [28–30]. Therefore, this study aimed to identify the differences in the structure of the gut microbiota between IgAN and controls and to evaluate the efficacy and mechanism of probiotics in the treatment of IgAN.

## Methods

### Patients

A total of 35 patients with IgAN diagnosed by renal biopsy at West China Hospital of Sichuan University between September 2018 and September 2020 were enrolled (IgAN group). Twenty-five healthy volunteers (HC group) were matched according to sex, age, and body mass index (BMI). Approximately 4 g of fresh feces from each subject was collected in a sterile container and immediately stored at − 80 °C. This study was conducted based on the Declaration of Helsinki and was approved by the Biomedical Ethics Committee of West China Hospital (ethical approval number: 2021-397). All included subjects signed an informed consent form at enrollment. The inclusion and exclusion criteria are shown in Additional file 1: Table S1.

### Animals and induction of IgAN

NLRP3^−/−^ (purchased from Jackson Laboratory, B6.129S6-Nlrp3tm1Bhk/J) mice on a C57BL/6 background and wild-type C57BL/6 mice were housed in our specific pathogen-free facility. Male mice aged 6–8 weeks were cohoused under the same conditions for two weeks prior to induction of IgAN to control for cage effects. The animal care and experimental protocol were approved by the Animal Ethics Committee of West China Hospital of Sichuan University.

IgAN was induced with bovine serum albumin (BSA), tetrachloromethane, castor oil, and lipopolysaccharide (LPS) for 8 consecutive weeks. This method of establishing the IgAN model has been widely reported, and our study utilized a slightly modified protocol (Fig. 2a). In short, 400 mg/kg BSA (Sigma, USA) dissolved in normal saline was administered intragastrically every other day for 8 weeks. A 0.15 ml mixture of tetrachloromethane and castor oil was injected subcutaneously every 7 days, with a gradually increasing proportion of tetrachloromethane. LPS (0.05 mg, Sigma, USA) was injected into the tail vein at the 6th week and the 8th week. The mice in the control group were treated via gavage, subcutaneous injection and tail vein injection of the same amount of normal saline at the same time points. After that, the mice were fed until the 13th week without any operation.

### Animal experiment design

#### Probiotic experiments

To investigate the efficacy of probiotics in the treatment of gut microbiota dysbiosis in an IgAN mouse model, wild-type mice were randomized to three groups: the (1) control wild-type mice (WC group), n = 6; (2) wild-type mice with IgAN (W-IgAN group), n = 7; and (3) wild-type mice with IgAN treated with probiotics (W-IgAN + B group), n = 6.

#### Mechanistic experiments

To assess the efficacy and mechanism of probiotics in the treatment of IgAN, wild-type and NLRP3^−/−^ mice were randomly divided into six groups: the (1) WC group, n = 6; (2) W-IgAN group, n = 7; (3) W-IgAN + B group, n = 6; (4) control NLRP3^−/−^ mice (NC group), n = 5; (5) NLRP3^−/−^ mice with IgAN (N-IgAN group), n = 5; and (6) NLRP3^−/−^ mice with IgAN treated with probiotics (N-IgAN + B group), n = 5.

#### SCFA experiments

Wild-type mice were randomly assigned to the following five groups to assess the influence of supplementation with SCFAs, which are metabolites of probiotics: the (1) WC group, n = 6; (2) W-IgAN group, n = 7; (3) W-IgAN + B group, n = 6; (4) wild-type mice with IgAN treated with sodium acetate (W-IgAN + SA group), n = 5; and (5) wild type mice with IgAN treated with sodium propionate (W-IgAN + SP group), n = 5.

### Probiotic and SCFA treatments

Probiotics mainly containing *Bifidobacterium longum* and *Lactobacillus bulgaricus* were purchased from Inner Mongolia Shuangqi Pharmaceutical Co., Ltd. Mice in the W-IgAN + B and N-IgAN + B groups were treated with 5,000,000 CFUs of *Bifidobacterium longum* and 5,000,000 CFUs of *Lactobacillus bulgaricus* by gavage every 2 days for 5 weeks. Mice in the control groups were given the same amount of normal saline intragastrically.

Sodium acetate (100 mM) and sodium propionate (100 mM) purchased from Sigma-Aldrich were dissolved in drinking water, which was refreshed three times a week; pH- and sodium-matched water was used as a control. This treatment lasted for five weeks, and mice in each group had ad libitum access to drinking water.

Except for intervention measures, all other conditions remained the same.

At the end of the 13th week, urine was collected and centrifuged at 3000 rpm for 20 min, and the supernatant was stored at − 80 °C. Fresh feces were put into a sterile microtube that was immediately placed in a liquid nitrogen tank for temporary storage. Blood was collected and the kidneys were harvested at 13 weeks. All the samples were stored at − 80℃ for subsequent testing.

### Bacterial DNA sequencing and bioinformatics analysis

Bacterial genomic DNA extracted from excrement samples was purified by using a DNA extraction kit (E.Z.N.A.^®^ Soil DNA Kit, Omega Bio-Tek, Norcross, GA, USA). The universal primer pair (338F/806R) spanning the V3-V4 hypervariable region of the bacterial 16S rRNA gene was used to amplify the bacterial genomic DNA on the Illumina MiSeq Platform (2 × 300 bp, Illumina, San Diego, CA, USA). Reads with 97% similarity were clustered into the same operational taxonomic units (OTUs). The ribosomal database project (RDP) classifier algorithm (http://rdp.cme.msu.edu/) against the Silva (SSU123) 16S rRNA database with a 70% confidence threshold was used to analyze the taxonomy of each representative sequence of OTUs. Bioinformatics analysis was performed on the Majorbio Cloud Platform (www.majorbio.com). Alpha diversity, including the Sobs, ACE and Chao diversity indexes, and beta diversity, mainly using PCoA, were assessed to determine the community richness and diversity of the gut microbiota.

### Enzyme‑linked immunosorbent assay

Enzyme-linked immunosorbent assay (ELISA) kits (Shanghai Enzyme-linked Biotechnology Co., Ltd., China) were used to detect the levels of urine protein, urine creatinine, serum creatinine, serum tumor necrosis factor α (TNF-α), serum interleukin-18 (IL-18) and serum IL-1β. All operations were performed in strict accordance with the instructions.

### Histology

Formalin (4%)-fixed paraffin-embedded renal tissues were serially cut into 4-µm-thick sections and stained with periodic acid-Schiff (PAS) reagent (Solarbio Co., Ltd. China), according to the manufacturer’s instructions. The stained slides were observed under an upright microscope (Carl Zeiss Meditec AG, Germany). Glomerular extracellular matrix was defined as the PAS-positive area quantified by image analysis software (ImagePro Premier 9), and the fraction of mesangial matrix was quantified by dividing the PAS-positive area by the glomerular volume.

Renal specimens embedded in optimal cutting temperature (OCT) compound and stored at − 80 °C were sliced with a cryostat. Staining for IgA and type 1 collagen (collagen 1) was performed on 8 µm frozen sections blocked with phosphate buffered saline (PBS) containing 5% goat serum. The primary antibodies rabbit anti-mouse IgA (1:500, Bioss bs-0774R) and rabbit anti-mouse collagen 1 (1:100 Abcam ab34710) were applied, and the sections were incubated at 4 °C overnight. The sections were washed with PBS and then incubated with the corresponding secondary antibodies (1:200 Abcam ab150081) for 1 h. DAPI (Thermo Fisher Scientific Inc.) diluted 1:1000 was used to stain the samples, which were then mounted with a cover clip containing anti-fluorescence quenching agent. Images were captured by confocal laser microscopy (Nikon Co., Japan). ImageJ 6.0 software (National Institutes of Health, Bethesda, MD, USA) was used to obtain the average fluorescence intensity (mean gray value) of the fluorescence channel.

### Western blot analysis

Kidneys harvested at the indicated times were homogenized in lysis buffer with protease inhibitors (Roche 4,693,159,001 and Roche 4,906,845,001). Total protein, measured with a bicinchoninic acid (BCA) protein kit (Beyotime Biotechnology Inc., China), was resuspended at 5 mg/ml. Total protein samples of the same volume and concentration were electrophoresed in SDS-PAGE gels and transferred to polyvinylidene difluoride membranes. Antibodies against NLRP3 (CST 15,101), Caspase 1 (Huaan ET1608-69), ASC (AdipoGen AG-25B-0006) and GAPDH (Zhongshan TA-08) were used after the membranes were blocked with 5% nonfat milk in Tris-buffered saline–Tween 20 (TBST) buffer, followed by incubation with goat anti-rabbit IgG H&L and goat anti-mouse IgG H&L (Zhongshan Corp., Beijing, China). The bands were visualized with a Bio-Rad ChemiDoc™ MP, and the intensity of the images was quantified using ImageJ 6.0 (National Institutes of Health, Bethesda, MD, USA).

### Quantitative Polymerase Chain Reaction (qPCR)

Total mRNA from the renal tissues was extracted with total RNA purification kits (Vazyme RC112), and the whole process was carried out in an RNase-free biological safety cabinet. A NanoDrop Lite spectrophotometer was then used to measure the RNA concentration and purity (OD260/280), and 2 µg of total RNA was reverse transcribed to cDNA using an RT-PCR kit (Vazyme R223-01). The PCR-specific primer pairs are presented in Additional file 1: Table S2. Complementary DNA was amplified in 2 × ChamQ SYBR Color qPCR Master Mix (Vazyme Q411-02/03). The relative expression of the target transcripts was normalized to the expression of endogenous control GAPDH and was calculated with the 2^−ΔΔCt^ method.

### Statistical analyses

The data are expressed as the mean ± standard deviation. Significant differences were assessed using a t test, one-way ANOVA, the Mann–Whitney U test or the Wilcoxon rank-sum test. The relationship between clinical manifestations and each bacterium at the genus level was assessed by Spearman's rank correlation. SPSS 26.0 software was used, and P < 0.05 (two-tailed) was considered statistically significant. Six microphotographs per group were used to perform area quantification in the immunofluorescence analysis. At least 3 kidney samples per group were used for western blot analysis and 6 renal tissues per group were used for qPCR.

## Results

### Intestinal dysbiosis featuring a reduction in *Bifidobacterium* is associated with IgAN patients

According to the strict inclusion and exclusion criteria, our study included 35 patients with IgAN and 25 healthy volunteers. There were no significant differences in sex, age, or BMI between the healthy control group (HC) and the disease group (IgAN). The blood pressure, serum creatinine level, urine protein concentration and number of urine red blood cells in the IgAN group were significantly higher than those in the HC group (Table 1), which was consistent with the clinical situation.

**Table 1:** Baseline characteristics of the enrolled volunteers

Characteristics	HC	IgAN	P value
Numbers	25	35	
Age (years)	31.5 ± 5.4	33.5 ± 6.2	0.10
Male (%)	12 (48%)	17 (48.6)	1.00
BMI (kg/m^2^)	22.5 ± 2.1	23.3 ± 3.5	0.56
SBP (mmHg)	111.6 ± 6.2	125.1 ± 16.5	< 0.01
DBP (mmHg)	69.4 ± 3.2	85.3 ± 9.9	< 0.01
sCr (µmol/L)	61.0 (55.0–70.0)	80.0 (58.5–106.0)	< 0.01
eGFR (mL/min/1.73 m^2^)	116.9 (114.0–125.9)	94.1 (70.8–118.8)	< 0.01
Urine protein (g/24 h)	0.06 ± 0.03	0.98 ± 0.63	< 0.01
Urine RBCs (/HPF)	0 (0–2)	13 (5–60)	< 0.01

*HC* healthy controls, *IgAN* IgA nephropathy, *BMI* body mass index, *SBP* systolic pressure, *DBP* diastolic blood pressure, *sCr* serum creatinine, *eGFR* estimated glomerular filtration rate, *RBCs* red blood cells, *HPF* high-power field

**Fig. 1:**
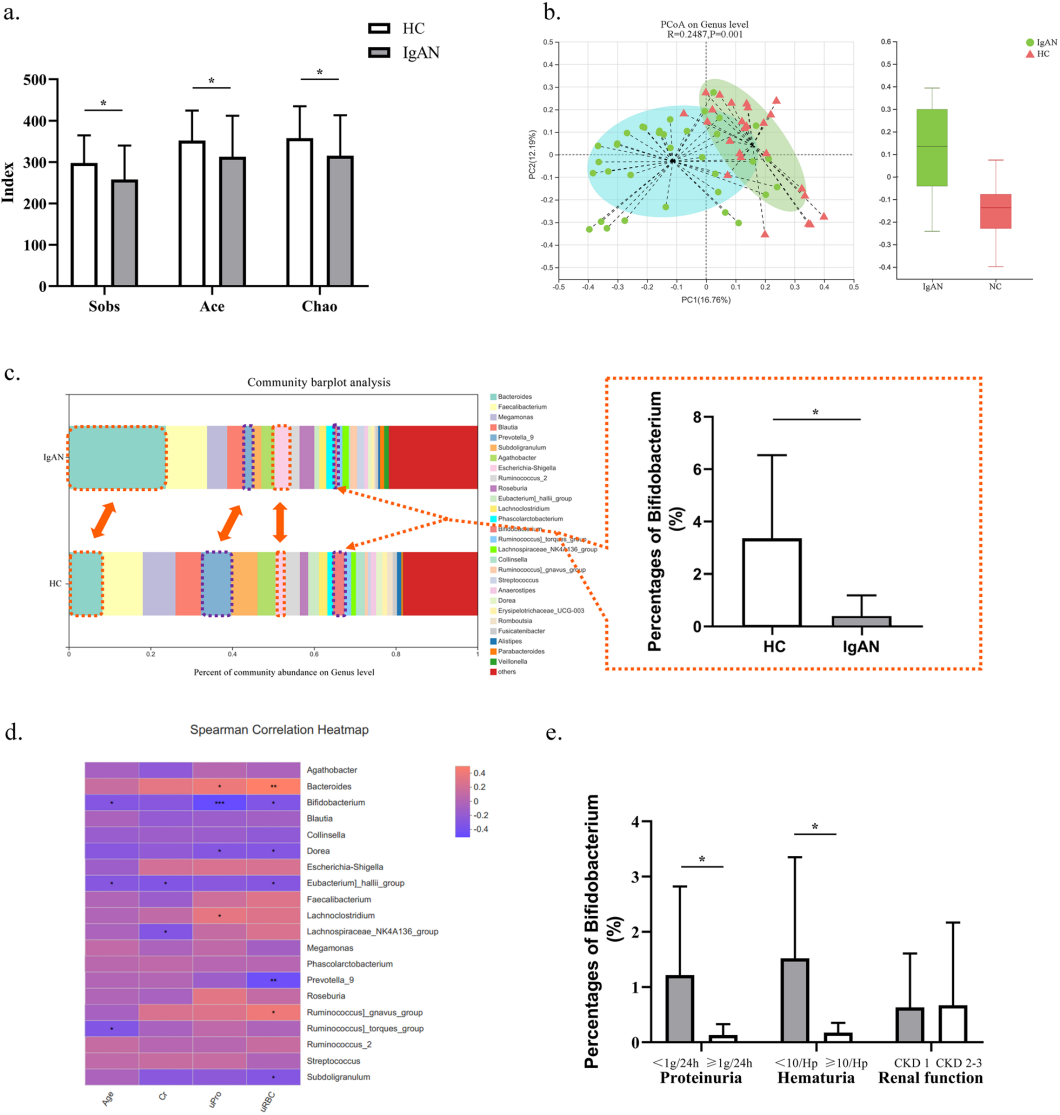
Intestinal dysbiosis was associated with IgAN patients. **a** The alpha diversity indexes of IgAN patients and healthy controls (HCs). **b** Beta diversity analysis using principal coordinate analysis (PCoA). **c** The community histogram of gut microbiota at the genus level and the *Bifidobacterium* proportions of IgAN patients and HCs. **d** Correlation heatmap analysis using the Spearman correlation coefficient to determine the relationship between intestinal bacteria and the severity of IgAN. **e** The relationship between *Bifidobacterium* and clinical manifestations of IgAN. * 0.01 < P ≤ 0.05. ** 0.001 < P ≤ 0.01. *** P ≤ 0.001

The alpha diversity was calculated to compare the community richness and diversity directly or indirectly between IgAN patients and HCs. The results suggested that the abundance diversity of gut microbiota in IgAN patients was significantly reduced compared with that of normal controls (Fig. 1a, P < 0.05). Beta diversity analysis also demonstrated that the overall structure of the intestinal flora differed significantly between the two groups (Fig. 1b). The above results supported the view that the intestinal microbial community structure in IgAN patients was altered.

A community histogram was used to demonstrate the proportions of different bacteria at the genus level, which clearly showed that the content of beneficial bacteria such as *Bifidobacterium* and *Prevotella 9* in the IgAN group was significantly decreased, while the abundance of *Bacteroides* in the IgAN group was significantly increased (Fig. 1c).

The Spearman correlation coefficient was presented through correlation heatmap analysis to determine the relationship between intestinal bacteria and the severity of IgAN. As shown in Fig. 1d, the abundance of *Bifidobacterium* was negatively related to age, urine protein levels and urine red blood cells. In addition, *Bacteroides* was found to be positively correlated with urine protein levels and urine red blood cells, while *Prevotella 9* and urine red blood cells had a negative correlation. The results indicated that the abundance of gut microbiomes was closely associated with the severity of IgAN.

Our study found that the percentage of *Bifidobacterium* in IgAN patients was 0.395 ± 0.794, which was much lower than that in healthy volunteers (3.103 ± 6.39, Fig. 1c). Moreover, subgroup analysis further confirmed the association between *Bifidobacterium* and IgAN. According to the degree of proteinuria, patients were divided into two groups: the mild proteinuria group (< 1 g/24 h, 17 individuals) and the moderate proteinuria group (≥ 1 g/24 h but < 3.5 g/24 h, 18 individuals). The results revealed that the proportion of *Bifidobacterium* in IgAN patients with moderate proteinuria was significantly less than that in patients with mild proteinuria (P = 0.013, Fig. 1e). Similarly, for IgAN patients in the moderate to severe hematuria group (≥ 10/Hp, 20 patients), the abundance of *Bifidobacterium* was markedly lower than that of patients with urine red blood cells < 10/Hp (P = 0.027). Notably, because the majority of the enrolled patients were in the early stage of CKD, the number of IgAN patients with renal failure was relatively small. Therefore, the patients were grouped based on CKD stages, and it was demonstrated that there were no significant differences in the abundance of *Bifidobacterium* among the different CKD stages.

### Probiotics could improve the structure of the intestinal flora in mice with IgAN

**Fig. 2:**
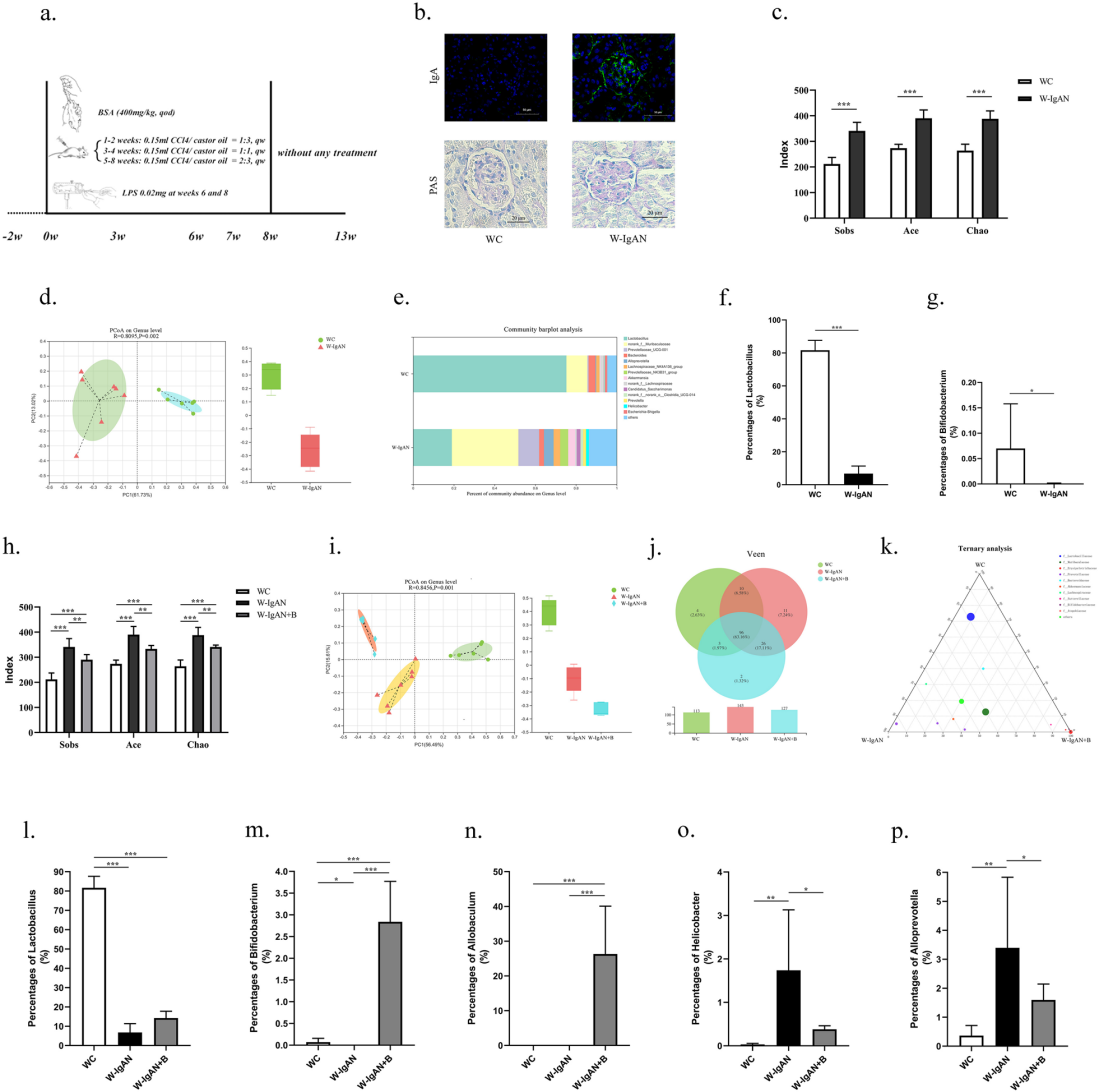
Probiotics could improve the structure of the intestinal flora in mice with IgAN.** a** The protocol for constructing an IgAN mouse model. **b** The renal pathology of wild-type mice (WC group) and IgAN mice (W-IgAN group) indicated that the IgAN model was successfully established. **c** The alpha diversity indexes of mice in the WC group and the W-IgAN group. **d** Beta diversity analysis of intestinal flora of IgAN mice and normal control mice. **e** Species composition analysis of IgAN mice and normal control mice at the genus level. **f**, **g** Comparison of abundance of intestinal *Lactobacillus* and *Bifidobacterium* in IgAN mice and normal control mice. **h**, **i** Analysis of alpha and beta diversity in IgAN mice treated with probiotics (W-IgAN + B group). **j**, **k** Venn diagram and ternary analysis of intestinal flora in IgAN mice treated with probiotics. **l**–**p** Comparison of the abundance of *Lactobacillus*, *Bifidobacterium*, *Allobaculum*, *Helicobacter* and *Alloprevotella* among the mice in the WC, W-IgAN and W-IgAN + B groups. * 0.01 < P ≤ 0.05. ** 0.001 < P ≤ 0.01. *** P ≤ 0.001

Animal studies were carried out in strict accordance with the experimental procedures (Fig. 2a). Specifically, 7 C57BL/6 mice were randomly assigned to the IgAN model (W-IgAN) group, and 6 mice were randomly assigned to the healthy control (WC) group. Renal pathology was used to confirm that the IgAN model was successfully established (Fig. 2b).

The feces from each group of mice were collected, and 16S RNA high-throughput sequencing was performed. Alpha diversity analysis indicated that the diversity of the gut microbiota in the W-IgAN group was different from that in the WC group (P < 0.001, Fig. 2c). Principal coordinate analysis (PCoA) revealed distinct differences in the intestinal bacterial community between the W-IgAN and WC groups (Fig. 2d). A genus-level comparison between W-IgAN and WC illustrated that the abundances of *Bifidobacterium* and *Lactobacillus* were sharply decreased, while the percentages of *Helicobacter* and *Alloprevotella* were significantly increased in the W-IgAN group (Fig. 2e–g).

Whether supplementation with probiotics could improve the intestinal bacterial community of IgAN aroused our interest. A five-week-long trial of probiotic intervention in 6 wild-type IgAN mice (W-IgAN + B group) was subsequently carried out. Probiotics mainly containing *Bifidobacterium* were used. The alpha analyses demonstrated that the community richness diversity indexes of the W-IgAN + B group, were between those of the WC and W-IgAN groups, suggesting that the structure of intestinal microbes in IgAN mice might be altered by probiotics (Fig. 2h, i). Both Venn diagram (Fig. 2j) and ternary analysis (Fig. 2k) demonstrated that the probiotics could improve but could not completely reconstruct the altered intestinal flora structure of IgAN.

Further analysis found that probiotics could increase the level of beneficial bacteria and significantly decrease the relative abundance of potentially pathogenic bacteria. The percentage of *Bifidobacterium* in the WC group was 0.0700 ± 0.0879, while *Bifidobacterium* accounted for 0.0006 ± 0.0013 in the W-IgAN group, which was far less than that of the normal control. The percentage of *Bifidobacterium* reached 2.8402 ± 0.9304 in the W-IgAN + B group (Fig. 2m), indicating that oral probiotics could significantly elevate *Bifidobacterium* levels in the intestine. However, oral administration of this type of probiotic did not significantly increase the *Lactobacillus* content in the IgAN mice (P > 0.05, Fig. 2l). Moreover, the relative abundance of *Allobaculum*, which had a beneficial effect on the host, was markedly enhanced by oral administration of probiotics to the IgAN mice (Fig. 2n). Conversely, probiotics competitively inhibited the growth of potentially pathogenic bacteria, including *Helicobacter* and *Alloprevotella* (Fig. 2o and p).

### Probiotics ameliorated IgAN by blunting the NLRP3 activating signal

Since IgAN was found to be related to the imbalance of intestinal flora and probiotic intervention could correct it to some extent, we speculated that probiotics might be helpful in the treatment of IgAN. IgAN mice, including both wild-type mice and NOD-like receptor pyrin domain containing 3 (NLRP3) inflammasome knockout (NLRP3^−/−^) mice, were given a 5-week intervention of probiotics to evaluate the effect and the mechanisms.

**Fig. 3:**
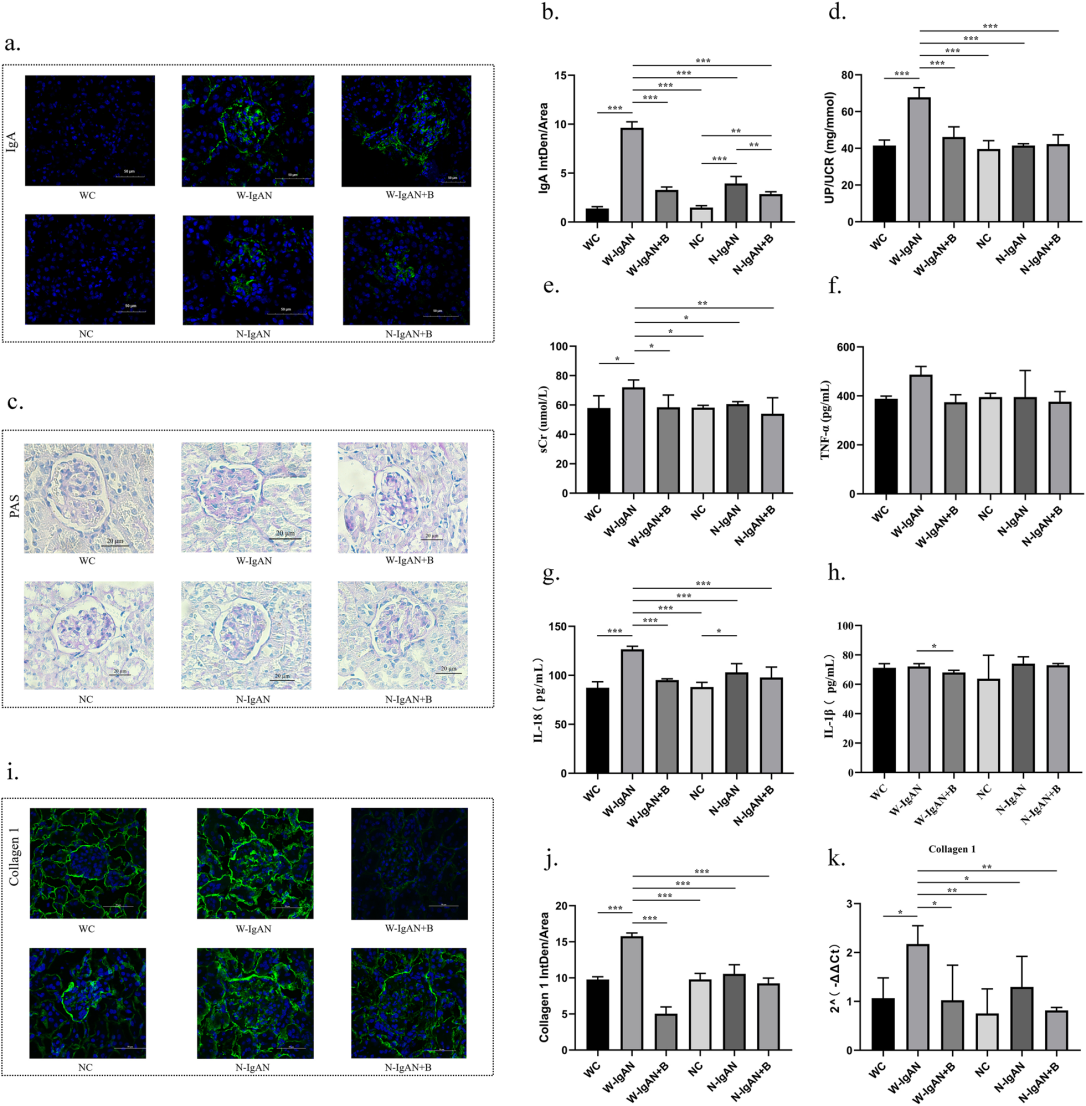
**a**, **b** Glomerular IgA immunofluorescence and the average fluorescence intensity of IgA in mouse kidney. **c.** PAS staining of wild-type normal controls (WC group), wild-type mice with IgAN (W-IgAN group), W-IgAN treated with probiotics (W-IgAN + B group), NLRP3^−/−^ mice (NC), NLRP3.^−/−^ mice with IgAN (N-IgAN), and N-IgAN treated with probiotics (N-IgAN + B). **d**, **e** The urinary protein/creatinine ratio and serum creatinine levels of the mice in different groups. **f–h** The serum inflammatory indicators of each group were measured by enzyme-linked immunosorbent assay. **i**–**k** The results of the immunofluorescence test and qPCR of collagen 1 in kidney tissue. * 0.01 < P ≤ 0.05. ** 0.001 < P ≤ 0.01. *** P ≤ 0.001

From Fig. 3a and b, it can be seen that hardly any IgA was deposited in the glomerular mesangium of the mice in the WC and NC groups, while the glomerular mesangial areas of the W-IgAN and N-IgAN groups had a large amount of IgA deposits, which was the same as the renal biopsy results of the IgAN patients and the healthy individuals. In wild-type mice, IgA deposition in the W-IgAN group was significantly increased, and probiotics significantly reduced the IgA fluorescence intensity. Similarly, in NLRP3^−/−^ mice, the number of IgA molecules in the N-IgAN group was also higher than that in the normal control group, and probiotics further reduced the IgA deposition of the N-IgAN group.

Periodic acid Schiff (PAS) staining (Fig. 3c and Additional file 1: Fig. S1) revealed that compared with those of the WC group, more mesangial matrix and mesangial hyperplasia were observed in the W-IgAN group, whereas probiotics could protect W-IgAN mice from mesangial proliferation. In contrast, the degree of mesangial hyperplasia in the N-IgAN group was not obviously different from that in the NC group and was significantly lower than that in the W-IgAN group. In addition, the mesangial lesions of the N-IgAN + B group did not change considerably compared with those of the N-IgAN group. Therefore, it could be speculated that both probiotics and NLRP3 gene knockout might reduce the mesangial proliferation of IgAN, but probiotics did not seem to be able to further improve the mesangial lesions of NLRP3^−/−^ mice with IgAN.

The urine protein-creatinine ratio of W-IgAN mice was markedly increased compared with that of WC mice (P < 0.001, Fig. 3d), which was consistent with the clinical manifestations of IgAN. Probiotics that mainly contained *Bifidobacterium* could significantly decrease the proteinuria-creatinine ratio of the W-IgAN group. Among NLRP3^−/−^ mice, the urine protein level of the N-IgAN group was not distinctly elevated, and probiotics could not further change the urine protein level of N-IgAN mice (P > 0.05), suggesting that the effect of probiotics on IgAN in NLRP3 gene knockout mice was not obvious. Similarly, the serum creatinine level in the W-IgAN group was slightly higher than that of the other groups (P < 0.05, Fig. 3e), implying that probiotics might decrease the serum creatinine level of wild-type IgAN mice, but it did not seem to change the serum creatinine concentration of NLRP3^−/−^ mice with IgAN.

The serum inflammatory indicators of each group measured by enzyme-linked immunosorbent assay (ELISA) are shown in Fig. 3f–h. These results indicate that the serum tumor necrosis factor-α (TNF-α) and interleukin-18 (IL-18) levels in the W-IgAN group were markedly higher than those in the WC group and that probiotics downregulated the expression of TNF-α and IL-18. However, there was no significant difference in the serum TNF-α concentration among the NC, N-IgAN, and N-IgAN + B groups. Although the IL-18 level of the N-IgAN group was higher than that of the NC group, the increase in the IL-18 concentration was far smaller than that in the W-IgAN group, and probiotics could not further reduce IL-18 levels in NLRP3^−/−^ mice with IgAN. Moreover, the serum IL-1β level in the IgAN groups was not significantly different from that of the control groups. Nevertheless, probiotics could still slightly reduce the serum IL-1β concentration of the W-IgAN group (P < 0.05, Fig. 3h).

The expression of collagen 1 in kidney tissue was used to reflect the degree of renal fibrosis. Figure 3i-k shows the results of the immunofluorescence test and qPCR. The results consistently revealed that the collagen 1 level of the W-IgAN group was visibly elevated and that probiotics could effectively downregulate the expression of collagen 1, while the level of collagen 1 in the N-IgAN group was much lower than that in the W-IgAN but was comparable to that in the NC and N-IgAN + B groups.

**Fig. 4:**
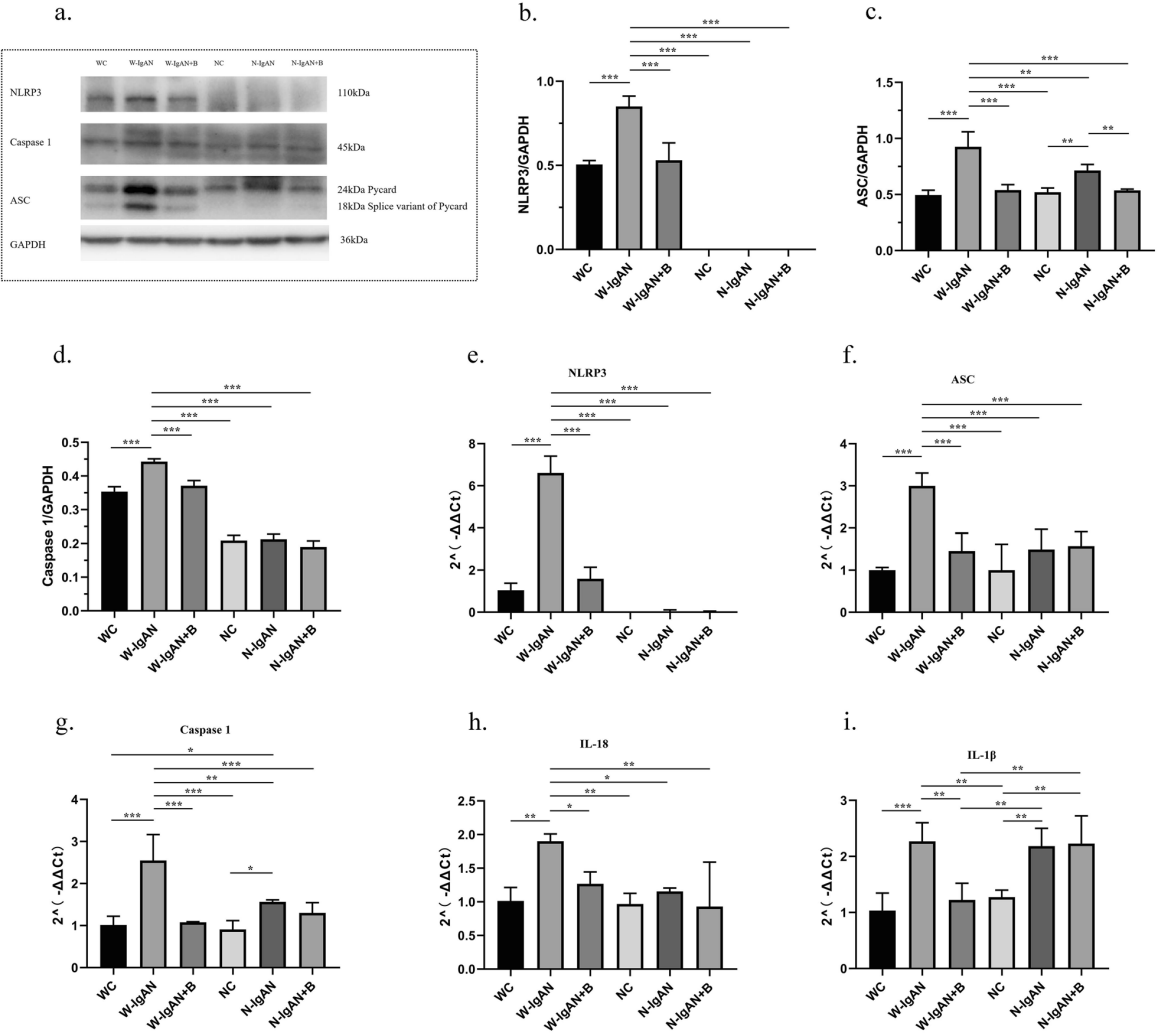
Probiotics could improve IgAN by inhibiting the NLRP3 signaling pathway.** a**–**d** The relative levels of NLRP3, ASC and Caspase 1 in mouse kidney tissues detected by western blot. **e**–**i** The mRNA levels of NLRP3, ASC, Caspase 1, IL-18 and IL-1β detected by qPCR. * 0.01 < P ≤ 0.05. ** 0.001 < P ≤ 0.01. *** P ≤ 0.001

To further confirm that probiotics could improve IgAN by inhibiting the NLRP3 signaling pathway, western blotting and qPCR were used to detect the relative levels of NLRP3, apoptosis-associated speck-like protein (ASC), and Caspase 1 in mouse kidney tissues. The protein and mRNA levels of NLRP3 in the W-IgAN group were significantly increased compared with those in the WC group, while the NLRP3 level in the W-IgAN + B group was equivalent to that in the WC group. Notably, the NLRP3 level was almost undetectable in all NLRP3^−/−^ mice, which verified the successful construction of our gene knockout mice (Fig. 4a, b and e). Moreover, the protein and mRNA levels of ASC and Caspase 1 were elevated in the W-IgAN group and could be decreased by probiotics and NLRP3 gene knockout in IgAN (Fig. 4c-d and f–g). Similarly, both probiotics and NLRP3 gene knockout reduced the expression of IL-18, but probiotics did not further inhibit the expression of IL-18 in the N-IgAN group (Fig. 4h). Although IL-1β is a downstream molecule of the NLRP3 signaling pathway, its mRNA expression was not significantly inhibited by NLRP3 gene knockout in our study. However, probiotics effectively altered its expression in the W-IgAN group (Fig. 4i).

### SCFAs decreased NLRP3 expression and protected against IgAN

**Fig. 5:**
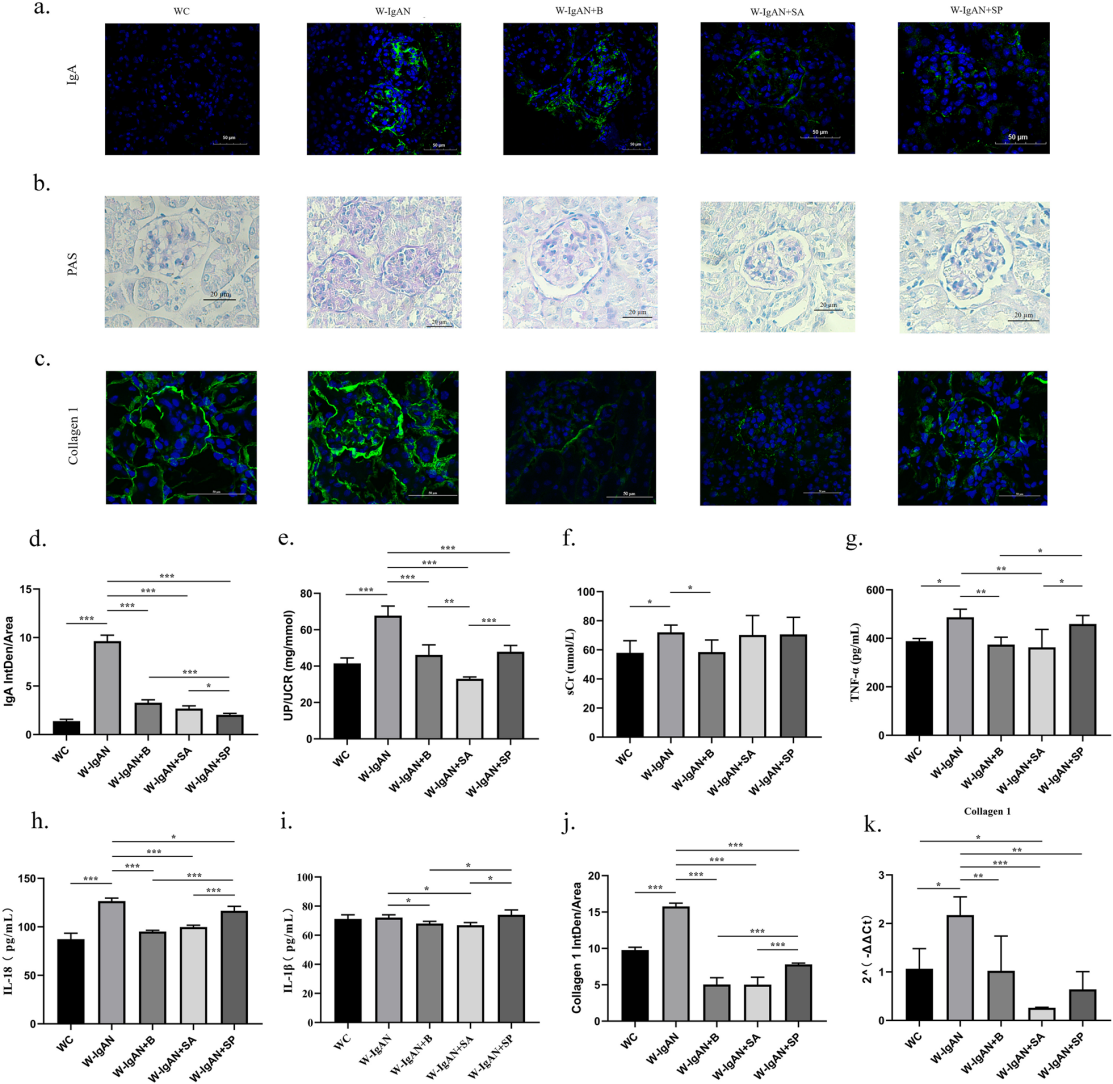
SCFAs provided significant renal protection and distinctly reduced urine protein levels, alleviated inflammation, inhibited excessive mesangial proliferation, and improved the degree of renal fibrosis in IgAN.** a** Glomerular IgA immunofluorescence in mouse kidney. **b** PAS staining of wild-type normal controls (WC group), wild-type mice with IgAN (W-IgAN group), W-IgAN treated with probiotics (W-IgAN + B group), W-IgAN treated with sodium acetate (W-IgAN + SA group) and W-IgAN treated with sodium propionate (W-IgAN + SP group). **c** Collagen 1 immunofluorescence in the mouse glomerulus. **d** The average fluorescence intensity of IgA in mouse kidney. **e**–**f** The urinary protein/creatinine ratio and serum creatinine levels of the mice in different groups. **g**–**i** The serum inflammatory indicators of each group were measured by enzyme-linked immunosorbent assay. **j** The average fluorescence intensity of collagen 1 in mouse kidney. **k** The relative gene expression of collagen 1 in mouse kidney tissue. * 0.01 < P ≤ 0.05. ** 0.001 < P ≤ 0.01. *** P ≤ 0.001

To further explore the renoprotective mechanism of intestinal probiotics, SCFAs (SA and SP), which are metabolites of *Bifidobacterium* and other probiotics, were used to treat IgAN mice. SA and SP effectively reduced the IgA fluorescence intensity of the W-IgAN group (Fig. 5a and d). Among them, SP worked the best, and its effect was superior to that of probiotics and SA. However, there was no significant difference in the efficacy of SA and probiotics. In addition, probiotics, SA and SP could distinctly improve mesangial hyperplasia of IgAN, whereas SA and SP seemed to have better effects than probiotics (Fig. 5b and Additional file 1: Fig. S2).

Both SA and SP significantly decreased the urine protein level of the W-IgAN group, the effect of which was equivalent to or better than that of probiotics (Fig. 5e). However, SCFAs might not be able to reduce the serum creatinine level of IgAN mice (Fig. 5f).

SA and probiotics could reduce the serum TNF-α and IL-1β levels, but there was no significant difference between the W-IgAN group and the W-IgAN + SP group, indicating that the TNF-α and IL-1β lowering effect of SP was not obvious (Fig. 5g and i). In addition, the serum IL-18 concentration in mice with IgAN could be significantly decreased by SA, SP and probiotics, but the effect of SP on reducing IL-18 levels seemed to be weaker than that of SA and probiotics (Fig. 5h). Therefore, SCFAs had the ability to reduce the level of inflammation in IgAN, but SA had a more substantial anti-inflammatory effect than SP.

Both collagen 1 protein and mRNA were distinctly downregulated by SA, SP and probiotics (Fig. 5j and k). Hence, it could be speculated that SCFAs might have the effect of improving kidney fibrosis. But the effect of SA on downregulating the protein and mRNA expression levels of glomerular collagen 1 was larger than that of SP.

**Fig. 6:**
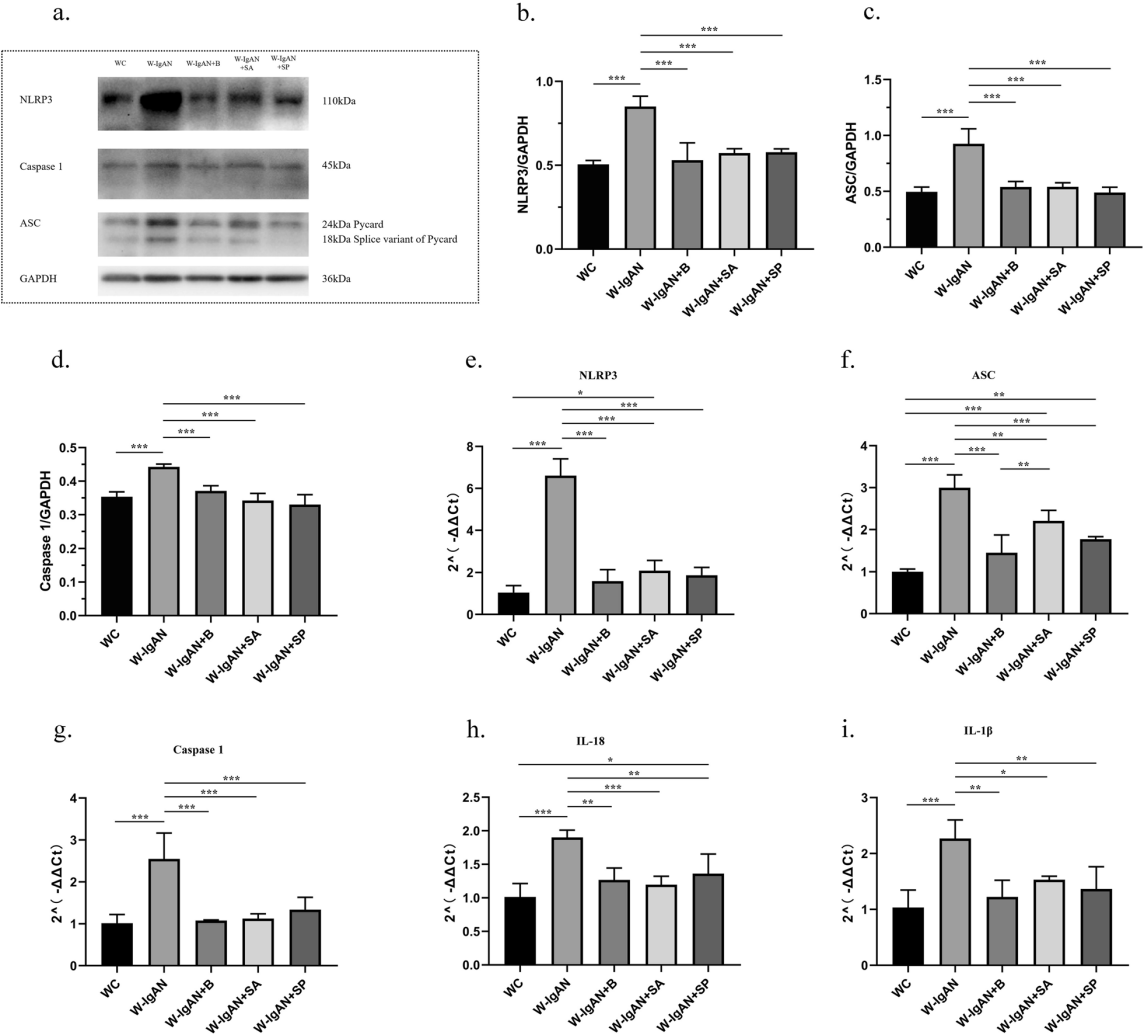
SCFAs significantly inhibited the NLRP3/ASC/Caspase 1 signaling pathway. **a**–**d**. The relative levels of NLRP3, ASC and Caspase 1 in mouse kidney tissues detected by western blot. **e**–**i.** The mRNA levels of NLRP3, ASC, Caspase 1, IL-18 and IL-1β detected by qPCR. * 0.01 < P ≤ 0.05. ** 0.001 < P ≤ 0.01. *** P ≤ 0.001

Western blotting was then used to detect the relative protein expression of NLRP3, ASC, and Caspase 1 in mouse kidney tissue to further investigate the mechanism by which SCFAs improved the manifestations of IgAN. Both SA and SP significantly inhibited the NLRP3/ASC/Caspase 1 signaling pathway, and both had the same effect as the probiotics (Fig. 6a–d). At the same time, qPCR was performed to confirm the results. Compared with expression in the W-IgAN group, the gene expression of NLRP3, ASC, Caspase 1, IL-18, and IL-1β in kidney tissues was significantly downregulated by SA and SP (Fig. 6f–i). Notably, the expression of ASC in the W-IgAN + SA group was slightly higher than that in the W-IgAN + B group, suggesting that SA had a weaker ability to downregulate ASC gene expression than probiotics, but this difference was not reflected at the protein level.

## Discussion

Current studies have confirmed that gut flora disorders are closely related to many immune-related diseases [7, 9]. Our study found that the dominant intestinal flora of patients with IgAN changed significantly. The levels of *Bifidobacterium* that is a common probiotic in healthy individuals and plays an essential role in human health, were decreased significantly. Another study involving the Italian Caucasian population found that IgAN might lead to changes in the structure of the intestinal flora, but the specific changes in the intestinal bacteria were different from our results, which might be due to genetic diversity, environment and eating habits [27]. Even so, both their group and ours found that the content of *Bifidobacterium* in the intestinal tract of IgAN patients was distinctly reduced. Additionally, our heatmap analysis and subgroup analysis revealed that *Bifidobacterium* was closely related to proteinuria and hematuria. It is worth mentioning that IgAN with a urine protein level greater than 1 g/24 h and persistent hematuria are independent risk factors for the progression of IgAN to end-stage renal disease (ESRD), implying that gut dysbiosis might be closely related to the progression of IgAN [31–33].

The physiology and anatomy of the gastrointestinal tract of mice and humans are very similar. However, the microstructure of the intestines of humans and mice is not the same, and the intestinal microbial communities are also different [34–36]. Although the differences in the gut microbiota between humans and mice cannot be ignored, our study found that IgAN patients and IgAN mice had gut dysbiosis and that both had a significant decrease in the abundance of *Bifidobacterium*. Most importantly, administration of probiotics containing *Bifidobacterium* could effectively improve the gut dysbiosis of IgAN mice, characterized by an increased the proportion of beneficial bacteria and a reduced abundance of potentially pathogenic bacteria. These results indirectly demonstrated that probiotics, especially *Bifidobacterium*, might be a new form of treatment.

After being stimulated by exogenous or endogenous factors, the NLRP3 inflammasome can activate ASC and Caspase 1 and then regulate the formation of IL-18 and IL-1β, causing an inflammatory cascade [22, 37]. Our study indicated that both probiotics and NLRP3 gene knockout significantly reduced IgA complex deposition and mesangial hyperplasia, effectively decreased the urine protein-creatinine ratio and serum creatinine concentration, obviously suppressed the expression of collagen 1 and greatly lowered the levels of proinflammatory factors. Notably, the effects of the probiotics and NLRP3 gene knockout were similar or equivalent. However, probiotic treatment seemed to have no effect on NLRP3^−/−^ mice with IgAN. Moreover, the protein and/or mRNA levels of NLRP3, ASC, Caspase 1, IL-1β and IL-18 were decreased by probiotic treatment. There is currently a lack of research on the mechanism of probiotics in the treatment of kidney disease, but a few studies have reported that probiotics, especially *Bifidobacterium*, could blunt the activating signal of NLRP3 in intestinal tissue. Sheng et al. used *Bifidobacterium* to treat mice with ulcerative colitis and found that *Bifidobacterium* intervention could significantly reduce the disease activity index and intestinal pathology scores and might significantly downregulate the TNF-α and IL-1β levels in colon tissue [24]. Yan et al. demonstrated that *Bifidobacterium* could exert anti-inflammatory effects by inhibiting the expression of the Toll-like receptor (TLR) 4, TLR9, NLRP3, and ASC genes, thereby reducing liver damage caused by a high-fat diet [38]. In addition, probiotics might also reduce the visceral hypersensitivity of irritable bowel syndrome by inhibiting NLRP3 [26]. These experiments indicated that *Bifidobacterium* might have a probiotic effect by inhibiting the NLRP3 signaling pathway. Therefore, it is reasonable to speculate that probiotics might improve the clinicopathological manifestations of IgAN by inhibiting the NLRP3 signaling pathway.

Previous studies have proved the benefits of probiotics in the scenario of gut diseases, in which it is reasonable to think that the benefits of probiotics are primarily local and, even, generated by physical contact between host and bacteria. In other words, probiotics colonize the gut, which is far from the kidneys, and the exact mechanism of how it exerts its renal protective effect is still unknown. More and more studies demonstrate that the concentration of SCFAs which are the main metabolites of probiotics, in chronic kidney disease, diabetic nephropathy and other diseases is significantly reduced. SCFA supplementation could improve kidney damage and have obvious renal protection. Therefore, we speculated that probiotics might act on renal tissue through the systemic circulation of its metabolites SCFAs, thereby improving the clinicopathological manifestations of IgA nephropathy.

Our study demonstrated that SA and SP provide significant renal protection and could distinctly reduce urine protein levels, alleviate inflammation, inhibit excessive mesangial proliferation, and reduce the degree of renal fibrosis in IgAN. SCFAs are produced by anaerobic bacteria and yeast fermentation in the colon from dietary fiber that has not been digested by the small intestine, and are then absorbed into the blood by the hepatic portal system [39]. SCFAs are important metabolites of probiotics and mainly exist in the human body in the form of SCFA salts, of which SA and SP are the most abundant components [15]. An increasing number of studies have shown that probiotic supplementation could increase the concentration of intestinal SCFAs, and SCFA supplementation could also increase the level of intestinal probiotics, suggesting that probiotics and SCFAs are inseparable [16, 21]. SCFAs are reported to be closely related to the health of the host and have direct or indirect effects on cardiovascular disease and kidney disease [15]. Currently, research on SCFAs in diabetic nephropathy and CKD is underway, but few studies have reported the efficacy of SCFAs in IgAN. A number of studies have found that serum SCFA levels in patients with CKD are significantly lower than those in healthy volunteers and that the administration of SCFAs could reduce the level of urinary toxins in patients and could delay the progression of the disease [17, 40–43]. SCFAs could also reduce the gene and/or protein expression of inflammatory cytokines, chemokines and profibrotic factors in the kidney tissue of diabetic mice and could protect diabetic mice from kidney disease [16]. However, the specific mechanism by which SCFAs exert renal protection is not fully understood. At present, some scholars have proposed that SCFAs might exert biological effects through the following two mechanisms: 1) SCFAs might inhibit histone deacetylases and modify histone tails to regulate epigenetic modification, and 2) SCFAs might also regulate G-protein-coupled receptor (GPCR) 41, GPCR 43, GPCR 109a and olfactory receptor 78 (Olfr 78) to play a biological role [16, 44–46]. Notably, our study showed that SCFAs might inhibit the NLRP3/ASC/Caspase 1 signaling pathway to improve the clinical symptoms and pathological damage associated with IgAN. GPCRs are transmembrane proteins that can recognize extracellular signals, transduce signals, act on the nucleus, and perform subsequent physiological functions [16]. NLRP3 is an intracellular protein that is mainly affected by nuclear factor kappa-B (NF-κB). Although related studies have reported that SCFAs might improve the inflammatory response by inhibiting the GPCR109a-NLRP3 pathway, the specific mechanism by which GPCR acts on NLRP3 is still unclear, and this may be an important direction for future research [47].

Moreover, our study also found that supplementation with probiotics (*Bifidobacterium longum* and *Lactobacillus bulgaricus*) could increase the content of *Allobaculum* in mice. Interestingly, the increase in the *Allobaculum* proportion was 10 times greater than that in *Bifidobacterium*. Emerging studies have reported that a decreased level of *Allobaculum* is correlated with a series of noncommunicable diseases, such as colitis, atherosclerosis, and polycystic ovary syndrome. Conversely, the increase in *Allobaculum* abundance was related to alleviation of these diseases [48–50]. Microbiota analyses revealed that the concentration of C18-3OH, the production of which could be one of the mechanisms implicated in the anti-inflammatory properties of probiotics, was correlated with an increase in the abundance in *Allobaculum *[48]. In addition, *Allobaculum* was reported to be a SCFA-producing bacterium [51]. Previous studies also found that probiotics (*Prevotella histicola*) protect against arthritis by increasing *Allobaculum* levles and augmenting butyrate production in humanized mice [48]. These findings implied that probiotics could increase the levels of other beneficial bacteria in the host. However, whether *Allobaculum* can be used to treat IgAN remains unknown, and should be verified in the future.

Our study has the following limitations. First, as a cross-sectional survey, we did not examine the relationship between microbiota imbalance and prognosis. Second, environmental factors, dietary habits and therapeutic drugs may affect gut microbial composition, and our results may apply only to the population of southwestern China. Third, the comparison of gut microbiota between IgAN and other glomerulonephritis was not initially included in this study, so it cannot be concluded whether the imbalance of gut microbiota is a characteristic manifestation of IgAN or all kidney diseases. Fourth, since most of the patients in our study were in the early stages of CKD (stages 1 and 2), further follow-up studies are needed to determine whether *Bifidobacterium* is associated with changes in renal function. Finally, although there are similarities in the gut structure and microbiota structure between mice and humans, the differences cannot be ignored. Therefore, whether the experimental results in mice are of clinical importance needs to be verified in the future.

## Conclusion

Substantial gut microbiota dysbiosis was present in IgAN, mainly manifesting as a significant decrease in the abundance of *Bifidobacterium*. The reduction in *Bifidobacterium* levels was strongly correlated with the severity of IgAN. Supplementation with probiotics mainly containing *Bifidobacterium* could markedly improve gut dysbiosis in IgAN. Moreover, both probiotics and their SCFA metabolites might attenuate the clinicopathological manifestations of IgAN by inhibiting the NLRP3/ASC/Caspase 1 signaling pathway.

## Supplementary Information

**Additional file 1: Table S1. **The inclusion and exclusion criteria of the clinical study. **Table S2.** The primer sequence of this study. **Figure S1.** PAS staining and statistical results of wild-type normal controls (WC group), wild-type mice with IgAN (W-IgAN group), W-IgAN treated with probiotics (W-IgAN+B group), NLRP3-/- mice (NC), NLRP3-/- mice with IgAN (N-IgAN), and N-IgAN treated with probiotics (N-IgAN+B). **Figure S2.** PAS staining and statistical results of wild-type normal controls (WC group), wild-type mice with IgAN (W-IgAN group), W-IgAN treated with probiotics (W-IgAN+B group), W-IgAN treated with sodium acetate (W-IgAN+SA group) and W-IgAN treated with sodium propionate (W-IgAN+SP group)

## Data Availability

Due to the privacy policy, the datasets analyzed in this study are not publicly available, but they are available from the corresponding author upon reasonable request.
