# Supplementary material for: Probiotics ameliorate IgA nephropathy by improving gut dysbiosis and blunting NLRP3 signaling
Source: J Transl Med. 2022 Aug 29;20:382. doi: 10.1186/s12967-022-03585-3 (PMC9422169; doi:10.1186/s12967-022-03585-3)
Supplement: Supplementary file 1 — Additional file 1: Table S1. The inclusion and exclusion criteria of the clinical study. Table S2. The primer sequence of this study. Figure S1. PAS staining and statistical results of wild-type normal controls (WC group), wild-type mice with IgAN (W-IgAN group), W-IgAN treated with probiotics (W-IgAN+B group), NLRP3-/- mice (NC), NLRP3-/- mice with IgAN (N-IgAN), and N-IgAN treated with probiotics (N-IgAN+B). Figure S2. PAS staining and statistical results of wild-type normal controls (WC group), wild-type mice with IgAN (W-IgAN group), W-IgAN treated with probiotics (W-IgAN+B group), W-IgAN treated with sodium acetate (W-IgAN+SA group) and W-IgAN treated with sodium propionate (W-IgAN+SP group) [file 12967_2022_3585_MOESM1_ESM.docx]

Supplementary Table 1. The inclusion and exclusion criteria of the clinical study.

| The inclusion and exclusion criteria | 1) The diagnosis of IgA nephropathy was in accordance with the KDIGO Clinical Practice Guideline for Glomerulonephritis. At least one experienced physician and one professional pathologist confirmed the diagnosis. Secondary IgAN such as lupus nephritis (Lupus nephritis, LN), hepatitis B associated nephritis (Hepatitis B associated nephritis) and Henoch-Schönlein purpura nephritis (Henoch-Schönlein purpura nephritis, HSPN) were excluded. |
| --- | --- |
|  | 2) The 24-hour urinary protein was less than 3.5g, and the estimated glomerular filtration rate (eGFR) was more than 30 mL/min/1.73 m2. |
|  | 3) All the enrolled individuals did not have diabetes, other autoimmune system diseases, liver function abnormalities, neuropsychiatric diseases, neoplasm, or kidney transplantation. |
|  | 4) All subjects had no obvious digestive system symptoms or diseases. Besides, no probiotics, antibiotics and prokinetic agents had been used in the past 4 weeks. |

| Gene | Forward (5' to 3') | Reverse (5' to 3') |
| --- | --- | --- |
| NLRP3 | TGCCTTGGGAGACTCAGGAG | CAGAGGTCAGAGCTGAACAACA |
| ASC | CTTGTCAGGGGATGAACTCAAAA | GCCATACGACTCCAGATAGTAGC |
| Caspase 1 | AATACAACCACTCGTACACGTC | AGCTCCAACCCTCGGAGAAA |
| IL-18 | GACTCTTGCGTCAACTTCAAGG | CAGGCTGTCTTTTGTCAACGA |
| IL-1β | GAAATGCCACCTTTTGACAGTG | TGGATGCTCTCATCAGGACAG |
| Collagen 1 | GGGCAAGACAGTCATCGAAT | ATTGGGGTGGAGGGAGTTTA |
| GAPDH | ACTTCAACAGCAACTCCCACTCT | GCTGTAGCCGTATTCATTGTCATA |

Supplementary Table 2. The primer sequence of this study.


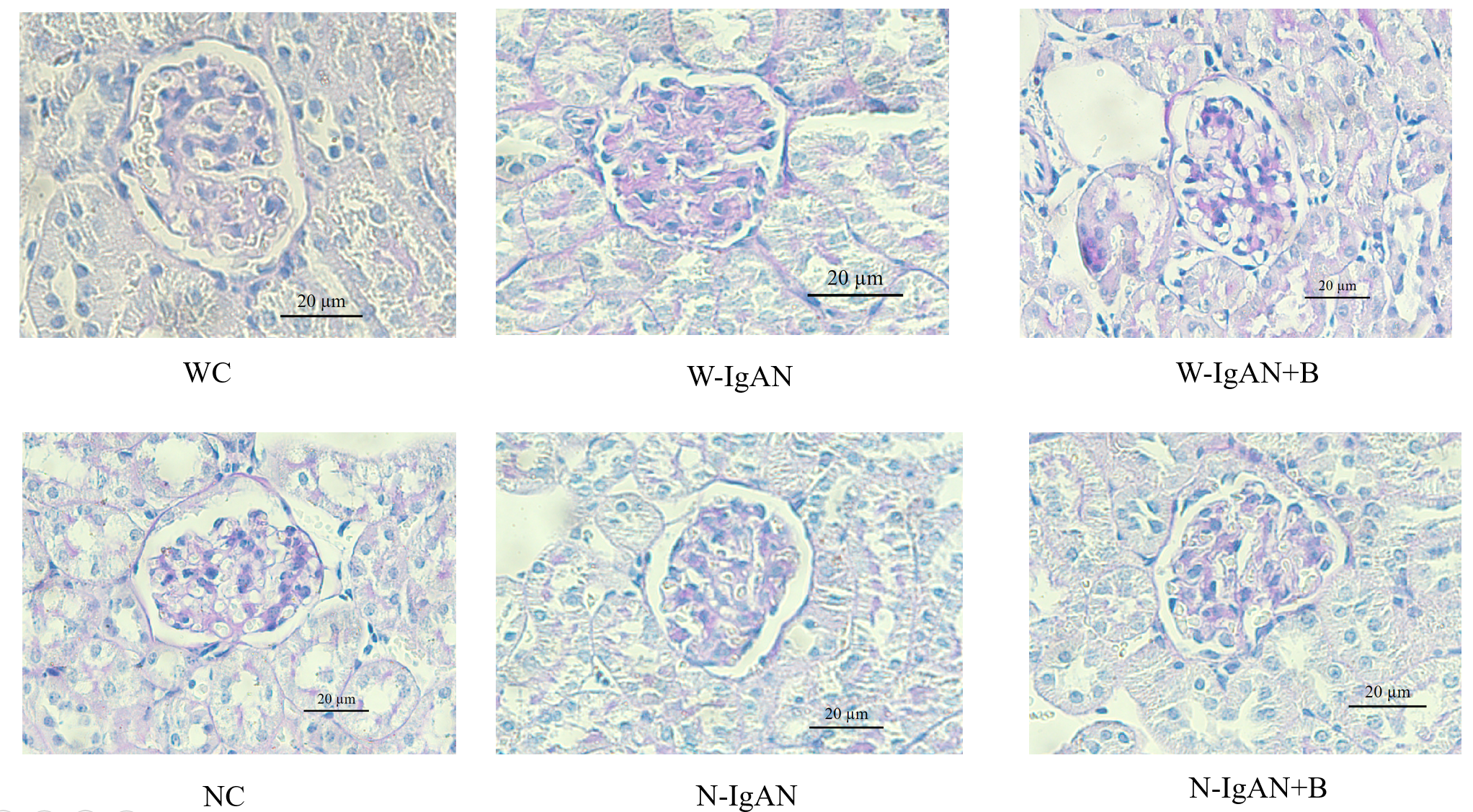


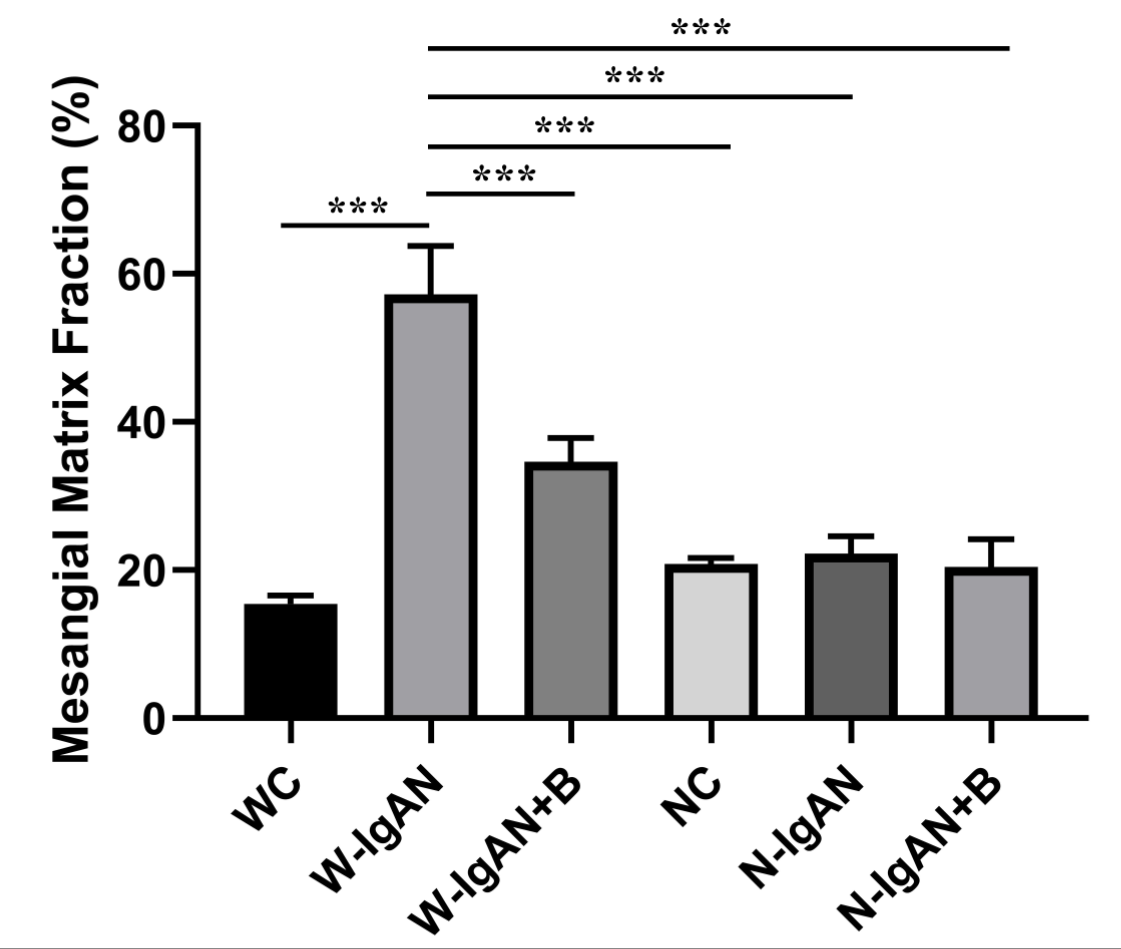


Supplementary Figure 1. PAS staining and statistical results of wild-type normal controls (WC group), wild-type mice with IgAN (W-IgAN group), W-IgAN treated with probiotics (W-IgAN+B group), NLRP3-/- mice (NC), NLRP3-/- mice with IgAN (N-IgAN), and N-IgAN treated with probiotics (N-IgAN+B).

* 0.01 < P ≤ 0.05

** 0.001 < P ≤ 0.01

*** P ≤ 0.001


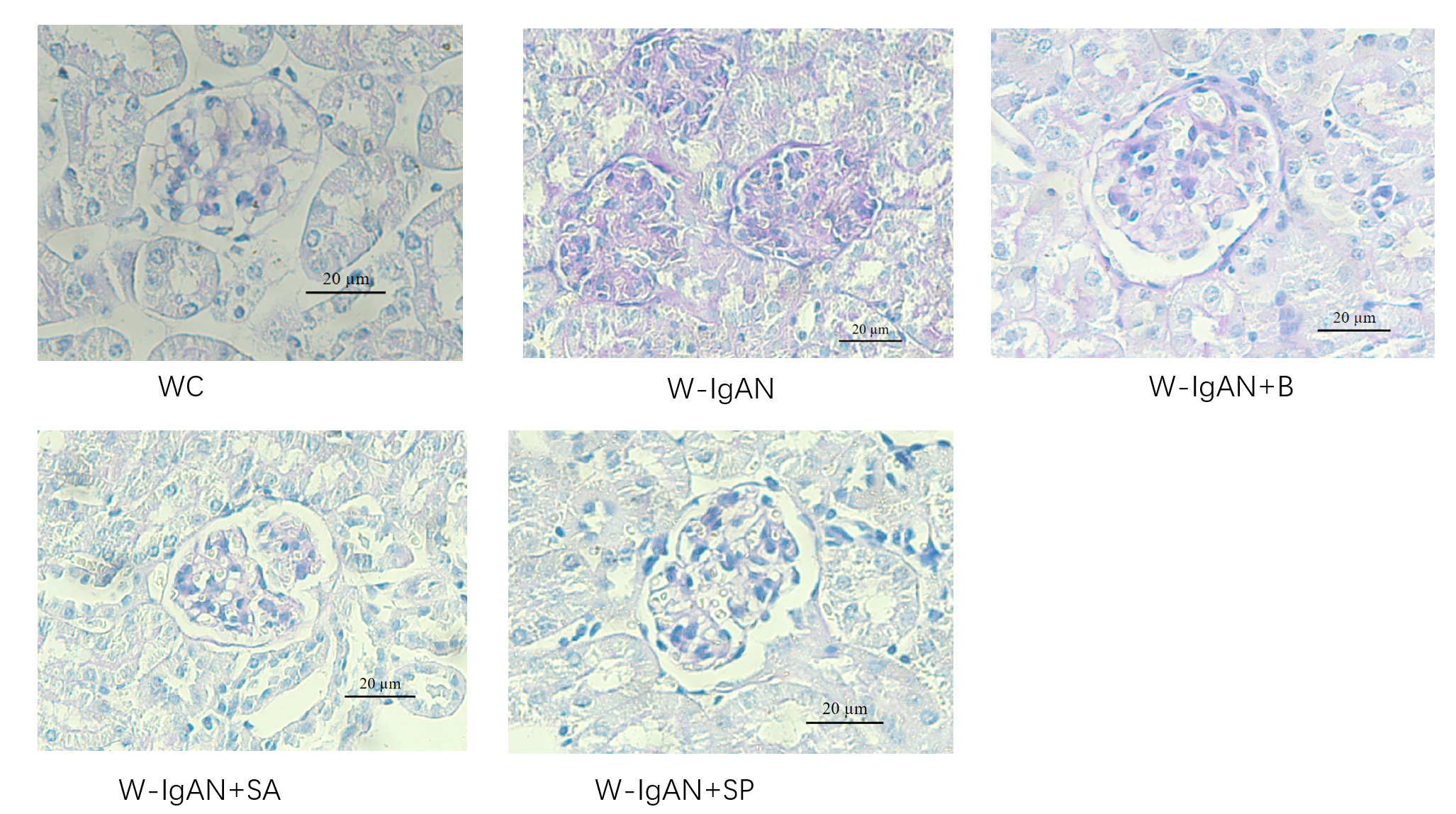


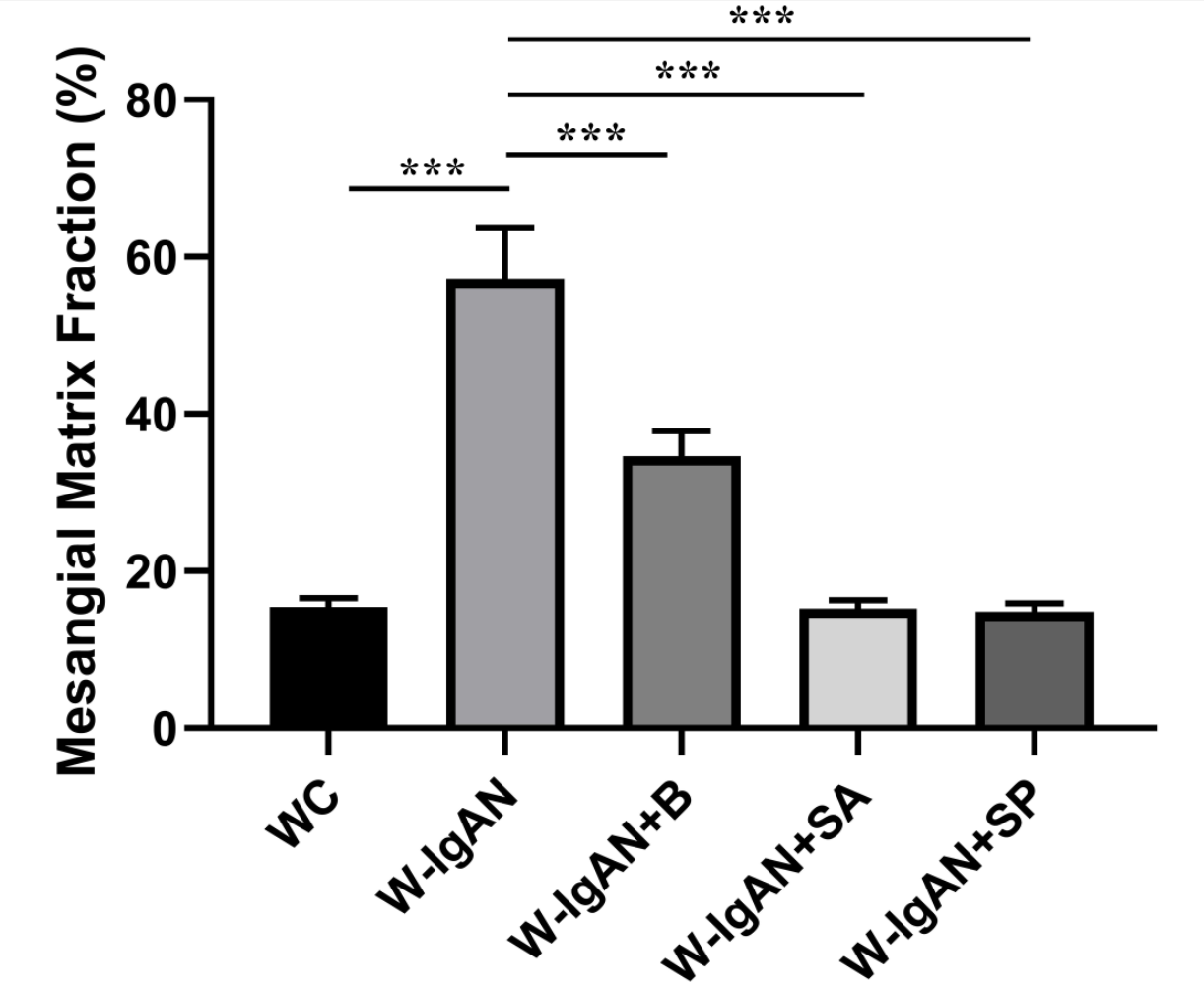


Supplementary Figure 2. PAS staining and statistical results of wild-type normal controls (WC group), wild-type mice with IgAN (W-IgAN group), W-IgAN treated with probiotics (W-IgAN+B group), W-IgAN treated with sodium acetate (W-IgAN+SA group) and W-IgAN treated with sodium propionate (W-IgAN+SP group).

* 0.01 < P ≤ 0.05

** 0.001 < P ≤ 0.01

*** P ≤ 0.001
